# The Study of Medicine

**Published:** 1829-07-01

**Authors:** 


### VIII.

**THE STUDY OF MEDICINE BY JOHN MASON GOOD, M.D. F.R.S. F.R.S.L. MEM. AM. PHIL. SOC. AND F.L.S. OF PHILADELPHIA. CONTAINING ALL THE AUTHOR'S FINAL CORRECTIONS AND IMPROVEMENTS. Third Edition, with much additional modern information on Physiology, Practice, Pathology, and the nature of Diseases in general. By *Samuel Cooper*, Surgeon to the King's Bench and Fleet Prisons; Surgeon to the Forces; Author of the Dictionary of Practical Surgery; Honorary Fellow of the Academy of Natural Sciences at Catania; &c. &c. In five vols. 8vo. London, 1829.**

[COMMENTARY THE FIRST.]

THE work before us may now be considered as a national one. It is highly improbable that any thing like a competitor can venture into the literary market for many years to come. The learning and research of Dr. Good, in medicine, and of Mr. Samuel Cooper, in Surgery, must deter even the most sanguine individual speculator from embarking in any similar undertaking for the next quarter of a century—and it does not seem probable that a joint-stock company, for the production of an "ENCYCLOPÆDIA MEDICA," shall soon start into existence in this country, notwithstanding the rage for this kind of adventure. No work in the English language has ever soared higher in the regions of medical literature and research than the "STUDY OF MEDICINE,"—and none, perhaps, has presented such astounding and unexpected chasms in modern pathology. Whether these chasms have been all filled up by the last emendations of the lamented deceased author, and the still more recent labours of the learned editor, will be seen as we proceed in our commentaries. As far as surgery is concerned, there can be no doubt; but it certainly does appear extraordinary to more than ourselves, that a physician was not associated in the editorial emendations. It is well known that we have always discountenanced the idea of there being any real or natural distinction between medicine and surgery; but custom having *forced* a distinction in practice, it is evident that the physician becomes more conversant with those diseases which are always under his care, than with those which are taken in charge by the surgeon; and although the surgeon meddles more with physic than the physician with surgery, yet it would be absurd to suppose that he studies purely medical cases with the same attention as the physician, or that he has the same field for observation. It is true that Dr. Good was himself a surgeon till within a few years of his death—but then he was a GENERAL PRACTITIONER, in which capacity his practice was ten times more medical than surgical. We grant too, that it would be difficult to pitch on any *pure surgeon* so well calculated for the task of a *medical* editor as Mr. Samuel Cooper; yet still we think that a prejudice will lie against the management,—whether well or ill founded, is another question. It will tend to diminish this prejudice when it is known that the original author had revised the whole work before his death—and that the present editor is peculiarly qualified to give to the surgical articles all the development of which they are susceptible. The same observation will apply to the anatomy and physiology of these volumes—but it will probably be shewn that the pathology of diseases, as apparent in this great work, has not been brought to the level of our actual or supposed knowledge at the present day. Perhaps a degree of the same deficiency will be seen in the therapeutical portions:—we do not mean a deficiency of therapeutical agents—for Dr. Good has not been sparing in the number which he has placed on his bill of fare—but in the discrimination of the valuable from the valueless articles. We believe Dr. Good was never in extensive practice—and that his practice, such as it was, had been confined to a very limited field of observation, topographically speaking. He commenced medicine at a late period of life too—and all these circumstances, together with his ardent pursuit of philological and poetical studies, may readily account for the exuberance of literature and the meagreness of practical information (as far as his own person was concerned) in the splendid work before us. In this respect the "STUDY OF MEDICINE" forms a striking contrast with Dr. Parr's "MEDICAL DICTIONARY,"—every page of which discloses the experienced and observant practitioner—while there is not as much erudition in the two large quartos of the Exeter physician, as in one of the smallest octavos of the London Philologist. Had Dr. Parr possessed the *learning* of Dr. Good, or Dr. Good, the *knowledge* of Dr. Parr, their works would have been invaluable! But it is doubtful whether a union of the two qualifications is ever to be expected in the same individual. POPE indeed has invested MILTON with the united qualities of Homer and Virgil—but medical *knowledge* is only to be obtained by long and close observation of disease at the bed-side of sickness—while medical literature can only be cultivated to a high degree in the library.

We have characterised the work before us as a national one—and as such it can be perfected and kept in a state of perfection only by national contributions. Successive editions will be demanded—and it will be the duty of future editors to collect the scattered rays of knowledge, from time to time, and concentrate them in this luminous focus. It has occurred to us that we might contribute to the utility of the work by taking a survey of those portions of it which we conceive to be open to criticism, or behind the van of pathological or therapeutical science. In doing this we hold out an invitation to our brethren in general to contribute any observations or remarks which their experience may suggest on any points of doctrine or practice—which communications shall be acknowledged or not, according to the option of the contributor.

It is not our intention to notice the physiological, historical, or descriptive portions of this work. Physiology is so completely in its infancy, that criticisms would be merely heaping conjectures on conjectures. Neither need we notice the nosological arrangements which we consider in much the same light as poetry or other works of the imagination. We believe Cullen's system of nosology to be just as good as any that has preceded or succeeded it—and if this be the case we see no necessity for going to school again to learn a new set of hard names for those things that are familiar to us by their old titles. The first article which we shall notice in this work is the 7th species of genus V, order I, class I, viz:—

### LIMOSIS DYSPEPSIA.

Considering the importance of this complaint, and the number of volumes which have been written on it, the article INDIGESTION is developed in this work very imperfectly—and indeed leaves the pathology, etiology and treatment of the proteian malady several years behind the current of medical literature on this particular subject. With the exception of a few lines in reference to a doctrine of Dr. W. Philip, neither the standard work of that gentleman, nor the works of Paris, Uwins, Johnson, or other modern writers on indigestion, are ever laid under contribution, or even referred to, except in a foot note at the end of the article. If the author had been a man who, from great experience and practical investigation, was capable of taking a lofty stand, and producing an original monograph on the intricate subject of indigestion, he might have been justified in rejecting all authorities except his own. But in this article there are few original ideas. It is a compilation—and, *as such*, it should have exhibited a fair epitome of the doctrines and practices of modern as well as the older writers. It has not done so, and the article is consequently incomplete. The following passage exhibits the pathological doctrine of Dr. Good, as respects dyspepsia. It has also more tendency to originality than most other parts of the article.

"The grand proximate cause of the three preceding species is debility of the stomach, whence, among other evils, an impaired secretion of gastric fluid. In the present instance, the debility is not often confined to the stomach, but extends to the intestinal canal, and the collatitious viscera, as the mesentery, the spleen, and the pancreas, especially the liver, in which it most frequently commences ; and hence another cause of the great complexity of this disease.

"The debility, and indeed torpitude, of the intestinal canal is evident from the habitual costiveness which so peculiarly characterizes this affection. Whether this be direct or indirect, intrinsic or sympathetic, as harmonizing with the weakness of the stomach, it is not easy to determine : but nothing can be a stronger proof of the great inactivity of the intestinal tube, from whatever cause produced, than the feebleness of its peristaltic motion, notwithstanding the acrimonious matters that are so frequently diffused over its inner surface.

"The imbecility of the liver is equally obvious in most cases, from the small quantity of bile that seems to be secreted, or its altered and morbid hue, as evinced by the colour of the fæces, which, in some instances, are of an unduly dark, and in others of an unduly light tint ; and possibly from the inactivity of the intestines themselves, whose peristaltic motion is conceived by Dr. Saunders and other pathologists to be, in a great measure, kept up by its stimulus." Vol. I. p. 190.

From this pathology, then, we find *irritability* or *irritation* wholly excluded—though every one, at all familiar with the disease, knows that the mere debility of the stomach and intestines forms but one feature of the complaint, and that the least important one—or, at all events, the least distressing. The author, indeed, is inconsistent with himself in the very next page. While admitting, with Dr. Philip, that when sympathetic disease (as in the lungs or brain) has taken place, the original malady in the stomach generally subsides, Dr. Good remarks :—

"And not unfrequently we can very advantageously take a lesson from this peculiar march of nature ; and by exciting an *artificial irritation* in some neighbouring and less vital part, can draw off the *morbid action* into such quarter." 191.

Thus, then, the grand proximate cause was stated to be debility, and not a word was said of irritation; yet, in the next page, we find that one of the ways in which this *debility* may be cured, is to excite an *artificial irritation* in another part, and thus draw off *morbid action*, in other words, irritation or inflammation, from the stomach itself.

We see, from these passages, that there is a fundamental error in the pathology or proximate condition in indigestion, whether we take the views of Philip, Abernethy, Armstrong, Abercrombie, Paris, Uwins, or Johnson. It may be irritation, it may be inflammation—but, at all events, it is not simply debility, as laid down by the author of this work. The defect in the pathology necessarily leads to a most dangerous error in practice. The antiquated doctrine of debility has carried millions to their graves, and driven tens of thousands mad. It led to the farrago of stimulants and tonics, when the stomach was in a state of *irritability* as well as *debility*, instead of low unirritating diet and tranquillizing medicines.

The whole etiology of this multiform disease is comprised within somewhat less than 30 lines! We shall give it here.

"The common causes of this imbecility, whether confined to the stomach, or co-extensive with the associate viscera, may be contemplated under two heads, *local* and *general:* under both which they are still further resolvable into the two opposite extremes of deficient and excessive stimulation; and consequently into a divergency of any kind from that medium of excitement and activity upon which health is made to depend.

"The local remote causes are, a too large indulgence in sedative and diluting substances; as tea, coffee, and warm water, or similar liquids taken as a beverage; or an equal indulgence in stimulant and acrid materials, as ardent spirits, spices, acids, tobacco whether smoked or chewed, snuffs, a daily habit of distending the stomach by hard eating or drinking; or a rigid abstemiousness, and very protracted periods of fasting.

The general remote causes are, an indolent or sedentary life, in which no exercise is afforded to the muscular fibres or mental faculties. Or, on the other hand, habitual exhaustion from intense study, not properly alternated with cheerful conversation; becoming a prey to the violent passions, and especially those of the depressing kind, as fear, grief, deep anxiety, immoderate libidinous indulgence; and a life of too great muscular exertion. *Perhaps the most common of this latter class of causes, are late hours and the use of spirituous liquors.*" 194.

We much doubt the truth of the last sentence, which we have marked in Italics. Among the upper classes of society, where late hours are kept, there is no immoderate use of spirituous liquors—and among the lower classes, where gin, rum, or whiskey is taken to excess, late hours are out of the question. A pretty extensive range of observation would induce us to place *moral emotions* at the very head of the list of remote causes of dyspepsia—especially among the upper classes of society. And whoever has attentively examined into *causes*, even in the humblest walks of life, will find abundant sources of mental anxiety operating *there!*

We agree with the talented author, that under whatever shape the disease appears, we are to recommend a subtraction of the *causes*. Yes! It is very easy to tell a man to—

"Pluck from the memory a rooted sorrow"—

but it is not quite so easy for the physician to—

"Purge the foul bosom of that perilous stuff
That weighs upon the heart."

Over the *physical* causes, indeed, of dyspepsia the patient has considerable command, under the guidance of a judicious practitioner; but over the *moral* causes he can exert but trifling sway!

One of the most lively passages in this article is that on watering places, as residences for those who have suffered from protracted study.

"It is to characters of this kind, perhaps, more than to any other, that the amusements of a watering place promise ample success; where the general bustle and hilarity, and the voluntary forgetfulness of care, the novelty of new scenes, and new faces, and new family anecdotes, and the perpetual routine of engagements that fill up the time with what would otherwise be trifles and frivolities, reverse the mischievous order and monotony of the past, break the sturdy chain of habit and association, and give leisure to the worn-out sensory to refresh itself." 195.

To this passage we see no objection; but we are by no means convinced of the solidity of the next precept laid down by the author, when the victims of a "town life of fashionable follies," are recommended to "a *total retreat from the world.*" But let Dr. G. speak for himself.

"Where the same effect has proceeded from a town-life of fashionable follies and dissipation, nothing is more common than to recommend a like change of residence. But in this case, though it may be a change of residence, it is not a change of life; and hence it is too often made without any benefit whatever. *A total retreat from the world*, the unbroken seclusion of a remote hamlet, the sober society of a few intimate friends, simple meals and early hours; instead of close and heated rooms, crowded and motley routs, costly feasts and midnight madrigals, are what are specially called for in this instance. 196.

The author properly remarks, that "the habitual use of hard eating and drinking must give way to a wholesome plainness of diet," though he is afraid, that "not a little mischief has often ensued from rigidly compelling the man who is suffering from a long habit of the former, to *abandon this habit at once*, and to run to an extreme of abstemiousness." Now is it not wonderful that the acute mind of Dr. Good should not have seen that, in the very same page, he recommends the patient to run into the most opposite extreme that can be imagined—"a total retreat from the world," after long habits of "motley routs and midnight madrigals?" The inconsistency here is very glaring—and the advice is not very judicious. Our author says:—"A spare diet, though often recommended, is rarely found of service, and very generally adds to the disease." But Dr. Good seems not to be acquainted with the severe forms of dyspepsy, where the sensibility of the stomach is so much augmented, that a spare diet at the beginning is essential to the cure. When, indeed, the stomach is able to bear the presence of food and drink without inconvenience, and to dispose of it without much difficulty, there can be no possible objection to fuller diet; but, till then, we apprehend that a spare diet will be found the best preparative regimen.

The remainder of the article is occupied with common-place remarks on bitters and tonics—but no where do we observe any enlightened view taken of this highly important disease. Its inflammatory or morbidly-irritable character is passed over by Dr. Good without notice—and the Editor only appends a few remarks from Drs. Abercrombie, Annesley, and Armstrong, on inflammation and organic changes in the stomach. We are of opinion that Mr. Cooper could have made great and useful additions to this article —and that he might have quoted, with more advantage to his readers, from recent medical writers than from the Edinburgh Review. It may be very witty and diverting in the Edinburgh reviewer, to deal out satirical strokes at the doctors about the "simplicity of savages"—"Prince Rasselas"—"Nebuchadnezzar"—the "flesh-pots in our kitchens," &c.; but we question the wisdom of introducing such non-professional irony in the midst of a grave, systematic work on medicine. We are disposed to think Mr. Cooper's good sense will induce him to replace this passage from the Edinburgh Review, in the next Edition, by some more solid instruction from the stores of medical literature, to which he has ready access, and from which he is so dextrous in gleaning the wheat from the chaff.*

---

* In justice to Mr. Cooper, we shall here insert a portion of the passage to which we have alluded above.

"The systems of dietetics offered to the world are innumerable, and marvellously contradictory to each other. Some, looking with an evil eye on the refinements of society, would bring us back to the simplicity of savages, and have us live 'according to nature.' Though, when we ask, with the Prince Rasselas, what it is to live according to nature, we are sure to meet with no more satisfactory answer, than what was vouchsafed to that noble inquirer. The truth is, however, that our bodies would be as little bettered as our minds, by going back to the state of savages; for it is now ascertained that savages are universally short-lived, and subject to sudden and violent diseases. Population increases slowly amongst them, and the healthiest and strongest of them, if compared with the average of well-fed civilized Europeans, will be found inferior both in strength and health. Some theorists again would have us live solely on animal food, and assert, that the human viscera bear vegetables 'only in a grumbling way;' while others would reduce us to the diet of Nebuchadnezzar, and not leave a flesh-pot in our kitchens. The different notions on dietetics by no means end here. Some sage doctors will never allow us to fill our stomachs, and some hold that they should never be altogether empty; some reduce the whole mystery of nutrition to a skilful exhibition of successive stimulants, and others to the exclusion of all that can interfere with the balsamic simplicity of the insipid chyle; some hold all fermented substances pernicious, and others think fermentation the best preparative for digestion. But as the judicious critic, to whom we are indebted for the above reflections, has observed, how is it possible to say what is absolutely the best diet for human beings, when we consider under what an infinite variety of different habits such beings are found to live in health and vigour, and by how many opposite causes their health and vigour are impaired? The same diet that is sanative to one, whose digestion has been weakened by scanty and penurious living, cannot possibly be suitable to another, who has suffered from a long course of repletion and excess. The regimen that is most wholesome for youth is not likely to be well fitted for old age; nor can that which answers for the active and laborious be proper for the studious and sedentary. Nay, your dry and adust subject plainly requires a different regimen from that of the plump and succulent. A lover should not be dieted as a miser; nor a champion of the fancy like a prime singer at the opera. Every man differs from every other in some of the important attributes of age, habit of body, occupation, temperament and disposition, to which may be added climate; so that all rules of diet must plainly require innumerable modifications to accommodate them to the condition of those classes of persons, even if it were possible to reduce them to certain classes." 214

